# Dacin, one metalloproteinase from *Deinagkistrodon acutus* venom inhibiting contraction of mouse ileum muscle

**DOI:** 10.1186/s12858-017-0086-0

**Published:** 2017-07-12

**Authors:** Bin Zhou, Gang Liu, Qiyi He, Bo Li, Xiaodong Yu

**Affiliations:** 1 Animal Toxin Group, Chongqing Key Laboratory of Animal Biology, Chongqing Engineering Research Center of Bioactive Substance, Engineering Research Center of Active Substance and Biotechnology, Ministry of Education, Collaborative Innovation Center of Breeding and Deep Processing of Venomous Snakes, College of Life Science, Chongqing Normal University, Chongqing, 401331 China; 2 Library, Chongqing Normal University, Chongqing, 401331 China

**Keywords:** Dacin, *Deinagkistrodon acutus*, Snake venom, Contraction tension, Mouse ileum

## Abstract

**Background:**

Mice were bitten by five-pace vipers (*Deinagkistrodon acutus*), and then envenomed. It was well-known that the snake venom mainly disturbed the blood homeostasis of the envenomed victims. Ocassionally, we found that the venom of *D. acutus* could inhibit the contraction tension of mouse ileum, so in this study we aimed to identify the active component inhibiting the contraction tension of mouse ileum in the snake venom.

**Results:**

The active component inhibiting the contraction tension of mouse ileum, designated as Dacin, was isolated from *D. acutus* venom, purified to protein homogeneity and composed of a single peptide chain, about 23 kDa analyzed by SDS-PAGE, and 22, 947. 9 Da measured by MALDI-TOF-MS. Not only the results of its PMF blasted by Mascot indicated that Dacin may be one snake venom metalloproteinase (SVMP), but also the results of the biochemical and in-vivo assays as follow demonstrated that it was one SVMP: it cleaved Aα and Bβ chains, not Cγ of bovine fibrinogen within 1 h, and also hydrolyzed fibrin polymer; besides its fibrino(geno)lytic activities were strongly inhibited by *β*- mercaptoethanol, EDTA and EGTA; and it could induce a hemorrhagic reaction under the dorsal skin of mouse. In the isolated tissue assays, Dacin caused the concentration-dependent and time-dependent inhibitory actions on the spontaneous contraction tension of the ileum smooth muscle of mouse, and the inhibitory effects were irreversible.

**Conclusions:**

Taken together, for the first time one active component (Dacin, a SVMP) that irreversibly inhibited the spontaneous contraction tension of mouse ileum has been isolated and identified from *D. acutus* venom. The findings may provide not only a new insight for toxicological researches on SVMPs and venoms of the vipers, but also a reference for clinicians to treat the snake-bitten victims. However, Dacin’s inhibitory molecular mechanism will be further studied in the future.

## Background

The five-pace vipers (*D. acutus*) are endemic to Southern China and a few of areas in Northern Vietnam. They are partially responsible for the envenomed and deaths resulted from the snakebites in China [1, 2]. It was well-known that the venom of *D. acutus* caused the blood homeostasis disturbances and the tissue damage of the victims, characterized by swelling, ecchymosis, hemorrhage, and necrosis at the bite, sometimes extending to the systemic symptoms [3–5]. Recently, the analysis results of transcriptome of the venom gland cells of *D. acutus* indicated that *D. acutus* venom chiefly contained metalloproteinases, C-type lectin, serine proteases, bradykinin-potentiating peptide, PLA2 etc. and the metalloproteinases and serine proteases in the snake venom played the pivotal roles in envenoming of the victims [6, 7]. A lot of the physiological or biochemical assays also revealed that the snake venom metalloproteinases (SVMPs) and serine proteases (SVSPs) had strong hemorrhagic and fibrin(ogen)olytic activities, acted as prothrombin activators, inhibited platelet aggregation, and hydrolyzed many structural proteins including extracelluar matrix proteins [8–10]. Although some documents mentioned that the viper venoms had slightly limited neurotoxic activities [11–13], till now, it has not been reported that *D. acutus* venom may inhibit the contraction tension of mouse ileum.

Interestingly, while we used several venoms in our laboratory including *Bungarus multicinctus, Gloydius shedaoensis, D. acutus* etc. collected in China, respectively, to test their effect on the contraction tension of mouse ileum, it was accidentally found that the venom of *D. acutus* could inhibit the contraction tension of mouse ileum, which seemingly performed like the neurotoxic activities. So in this study in order to confirm or unravel *D. acutus* venom’s such the function or activity, we focused on the active component, which could inhibit the contraction tension of ileum, in the venom of *D. acutus,* and isolated, purified and identified it from the venom of *D. acutus.*

## Methods

### Snake venom and animals

Snake venoms were milked from *D. acutus* captured in Chongqing, China, and lyophilized for experimental use. Kunming mice (20 ± 2 g of body weight) were obtained from the Laboratory Animal Center of the Third Military Medical University. They were housed in temperature-controlled rooms and received water and food ad libitum until use.

### Reagents

Sephadex G-50, DEAE Sepharose Fast Flow and Hitrap Capto DEAE were purchased from GE Healthcare (USA). Protein MW Marker (Low) was obtained from TAKARA (Japan), ACN and Methanol from Fulltime Co. (China), and Bovine thrombin and fibrinogen from Biosharp (China). All other chemicals were of analytical grade.

### Preparation of mouse ileum tissues

The preparation method of ileum tissues was modified as described in several reports [14–16]. Mice were killed by cervical dislocation and a segment of ileum approximately 15 cm long was removed from a distance of 2 cm from the ileo-caecal junction and kept in Krebs’ solution (118.4 mM NaCl, 4.7 mM KCl, 1.2 mM MgSO_4_, 1.2 mM KH_2_PO_4_, 2.5 mM CaCl_2_, 25.0 mM NaHCO_3_, and 11.1 mM glucose, pH 7.4) oxygenated with 95% O_2_ and 5% CO_2_. The mesentery and fatty tissues were removed and the lumen carefully flushed of its content with Krebs’ solution. Segments of ileum approximately 2 cm in length were dissected and mounted vertically in 10 ml water-jacketed organ baths containing Krebs’ solution kept at 37 °C and oxygenated with 95% O_2_ and 5% CO_2_. Changes in tissue tension were measured isometrically using force displacement transducer (Biopac, USA) and recorded on MP36 system (Biopac, USA). The tissues were slowly placed under a resting tension of 0.5 g (unless otherwise stated) and allowed to equilibrate for an at least 20 min period before the construction of the agonist or antagonist concentration-response curves. The active tension and rate of spontaneous tensions were continuously monitored for up to 90 min throughout the experiment. To avoid tachyphylaxis caused by the repeated use of the same ileum segment in each experiment, the used ileum segment was replaced with new one [17]. In control experiments, the ileum segment was incubated with normal saline for at least 90 min without apparent decline in the parameters.

### Isolation and purification of protein component

*D. acutus* venom (200 mg) was dissolved in 2.5 ml of 0.05 M Tris-HCl buffer (pH 8.4) overnight at room temperature, and centrifuged at 5000 rpm for 10 min at room temperature. The supernatant was loaded on a Sephadex G-50 column (1.1 cm × 100 cm) equilibrated with 0.05 M Tris-HCl buffer (pH 8.4), then eluted with the same buffer at an elution rate of 0.15 ml/min. The isolated fraction with the strongest inhibitory contractile response of ileum muscle was loaded on a DEAE Sepharose Fast Flow column(1.6 cm × 20 cm) equilibrated with 0.05 M Tris-HCl buffer (pH 8.4), and chromatographed with a linear gradient of 0 to 0.2 M NaCl in 0.05 M Tris-HCl buffer (pH 8.4) at an elution rate of 1.5 ml/min. The obtained fraction was pooled, desalted and concentrated, then applied to a Hitrap Capto DEAE column (0.7 cm × 2.5 cm) pre-equilibrated with 0.05 M Tris-HCl buffer (pH 7.4), and chromatographed with a linear gradient of 0 to 0.8 M NaCl in 0.05 M Tris-HCl buffer (pH 8.4) at an elution rate of 1.5 ml/min. The final active peak was manually collected, then desalted, lyophilized and stored at −20 °C.

### Reversed-phase HPLC

The venom protein sample was applied to a C_18_ column (4.6 mm × 250 mm, ø 5 μm), and eluted using an acetonitrile-trifluoroacetic acid (TFA) gradient (buffer A: 0.1% TFA, buffer B: 80% acetonitrile-0.1% TFA; gradient: 0-30 min: 80% B, 30-35 min: 80–100% B) at a flow rate of 1 ml/min. The elution peaks were monitored at an absorbance of 215 nm. The major peak was collected and lyophilized for mass spectrometry and other studies.

### Protein concentration

Protein concentration was determined by the Lowry method [18] with BSA as a standard.

### SDS-PAGE

SDS-PAGE under reducing and non-reducing conditions were carried out according to Laemmli method [19].

### MALDI-TOF mass spectrometry

Protein masses were determined by Matrix assisted laser desorption/ionization time-of-flight mass spectrometry. Spectra were recorded and analyzed using an AB SCIEX instrument in a linear positive mode. The protein band of interest was sliced from 15% SDS-PAGE, and reduced, alkylated, then subjected to digestion with trypsin. The peptide mixtures were dried and analyzed with an ABI Voyager-DE Pro MALDI-TOF mass spectrometer. The peptide mass fingerprint (PMF) results were compared with the trypsin digest of protein of NCBInr database by using Mascot software 2.3.02.

### Fibrino(geno)lytic activity assay

The hydrolytic activities of the purified venom protein on fibrinogen were evaluated by SDS–PAGE according to Rodrigues et al. [20] with some modifications. Different amounts of the purified venom protein (0.4 μg – 2.4 μg), or different mixtures of 2.4 μg of the purified venom protein with the different inhibitors (0.05 M PMSF, 0.05 M *β*-mercaptoethanol, 0.05 M EGTA and 0.05 M EDTA, respectively), were separately incubated with 20 mL of 10 mg/mL bovine fibrinogen (0.05 M PBS, pH 8.0) at 37 °C for 1 h. All the reactions were terminated with 10 mL of Tris–HCl buffer (0.05 M, pH 8.8) containing 10% (*v*/v) 2-mercaptoethanol, 2% (*v*/v) SDS, and 0.05% (*w*/*v*) bromophenol blue. The final reaction mixtures were analyzed by SDS–PAGE gels (12%, *w*/*v*).

Fibrinolytic activity was measured on fibrin plate. Fibrin plate was made of 8 mL of 0.4% fibrinogen, 8 mL of 1% agarose and thrombin (20 U) in 0.025 M Tris-HCl buffer (pH 7.4). After the wells (3 mm in diameter) were made in the plate, an aliquot volume (15 μL) of saline, Dacin (8 μg) and crude venom (20 μg), respectively, were added into the wells, then incubated at 37 °C for 12 h to visualize the transparent zones.

### PLA_2_ activity assay

PLA_2_ activity was determined according to the methods reported by Habermann and Hardt [21] with some modifications. Briefly, one part of egg yolk was mixed with 3 parts of 0.85% (*V*/V) NaCl and centrifuged for 2 min at 2000 rpm, and the supernatant (egg yolk suspensions) was transferred into tubes for the following use. Agarose (0.15 g) was dissolved in 25 mL of 50 mM sodium acetate buffer (pH 7.5) in boiling water bath, then the solution was cooled down to 50 °C. The cooled agarose solution, egg yolk suspensions (500 μL) and 10 mM CaCl_2_ solution (250 μL) were fully mixed, finally was poured into Petri dishes. After the wells were punched in the plate, an aliquot volume (15 μL) of saline, purified venom protein (8 μg) solution and crude venom (20 μg) solution, respectively, were added into the wells, and incubated at 50 °C for 20 h to visualize the transparent zones.

### Hemorrhagic activity

According to the method [22], Kunming mice (18–20 g) received common feedstuff and water freely. To evaluate the hemorrhagic activity of purified venom protein, groups of 4 mice were injected intradermally on the dorsal region with the following dosages, respectively: a, 100 μL of 0.9% saline solution; b, 100 μL of saline solution containing 20 μg of *D. acutus* venom; c, 100 μL of saline solution containing 30 μg of purified venom protein; d, 100 μL of saline solution containing 10 μg of purified venom protein. Two hours after the injection the mice were sacrificed and the dorsal skin was sectioned for observation.

### Statistical analysis

Data analyses were performed using the PRISM 5.0 software package. The results regarding biological activities were presented as means and standard deviation. Statistical analysis of significance was carried out by one-way or two-way ANOVA, The value of *p* < 0.05 was considered significant.

## Results

### Protein purification

**Fig. 1:**
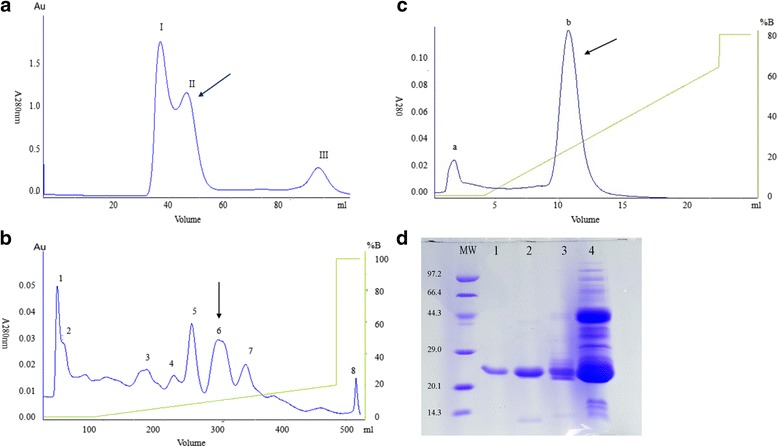
Chromatograms of Dacin isolated and purified from *D. acutus* venom and its SDS-PAGE identification. **a**, Chromatogram of 200 mg *D. acutus* venom on Sephadex G-50 column (1.1 cm × 100 cm), eluted with 50 mM Tris-HCl buffer (pH 8.4) at a rate of 0.15 ml/min. Peak II showed Dacin’s activity. **b**, Chromatogram of Peak II on DEAE Sepharose Fast Flow ion-exchange column (1.6 × 20 cm), eluted with 50 mM Tris-HCl buffer (pH 8.4) at a rate of 1.5 ml/min, then combined with a linear gradient NaCl (0-0.2 M) elution. Peak 6 presented Dacin’s activity. **c**, Chromatogram of Peak 6 on Hitrap Capto DEAE ion-exchange column (0.7 × 2.5 cm). Peak b exhibited Dacin’s activity. **d** 15% SDS-PAGE analysis of *D. acutus* venom and Dacin. MW lane, standard protein markers (kD); lane *1*, Peak b; lane *2*, Peak 6; lane *3*, Peak II; lane *4*, *D. acutus* venom

From *D. acutus* venom, an active component was isolated and purified to homogeneity through three-step chromatographies including Sephadex G-50, DEAE Sepharose Fast Flow and Hitrap Capto DEAE. By Sephadex G-50 chromatography, three peaks were obtained (Fig. 1a) and Peak II exhibited the inhibitory activity on the contraction tension of ileum. Peak II was further fractioned into eight peaks by DEAE Sepharose chromatography (Fig. 1b), among which only Peak 6 showed the inhibitory activity on the contraction tension of ileum. Finally, by Hitrap Capto DEAE chromatography Peak 6 was isolated into two peaks (Peak a and Peak b) (Fig. 1c). Only Peak b of both peaks presented the strong inhibitory activity on the contraction tension of ileum, and it showed single one protein band on SDS-PAGE (Fig. 1d), which was named as Dacin.

### Homogeneity and mass spectrometry analysis

**Fig. 2:**
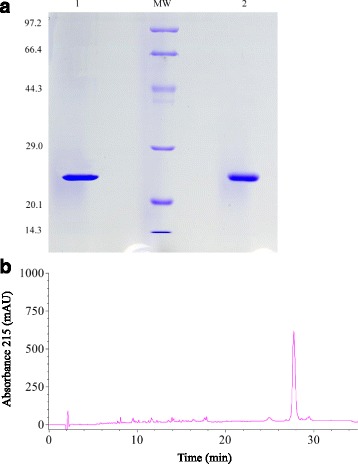
SDS-PAGE and HPLC analysis of Dacin. **a**, 12% SDS-PAGE spectrums of Dacin. Lane *1*, Dacin under reducing conditions; MW lane, standard protein markers (kD); lane *2*, Dacin under non-reducing conditions. **b**, Chromatogram of Dacin sample on a analytical C18 column of HPLC, equilibrated with buffer A (0.1% TFA) and eluted with the following gradient of buffer B (80% acetonitrile in 0.1% TFA) and buffer A at a rate of 1 ml/min

Either under reduced or non-reduced conditions, Dacin exhibited unique one band on SDS-PAGE (Fig. 2a). RP-HPLC also demonstrated that Dacin was fractioned into only one peak (Fig. 2b). Its molecular weight was about 23 kDa revealed by SDS-PAGE, and was 22,947.9 Da determined by MALDI-TOF-MS (data not shown). The PMF results of Dacin were searched in NCBInr database by Mascot software and it was found that Dacin highly matched Ac1-proteinase that is a SVMP in the venom of *D. acutus* from Taiwan [23].

### Fibrino(geno)lytic activities

**Fig. 3:**
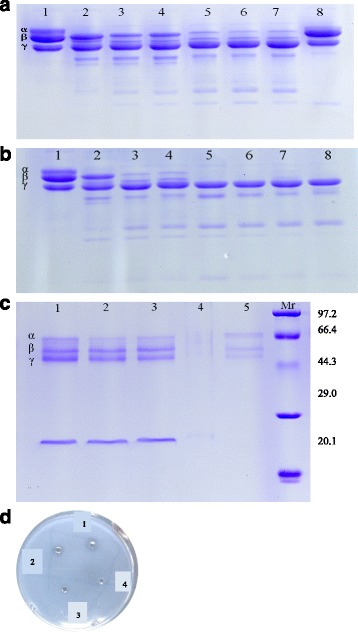
Fibrinogenolysis activity of Dacin analyzed by 12% SDS-PAGE and its fibrinolysis activity tested by fibrin plate assay. **a**, Effects of different concentrations of Dacin incubated at 37 °C for 30 min with 25 μg of bovine fibrinogen. Lane *1*, control; lane *2*, 0.4 μg; lane *3*, 0.8 μg; lane *4*, 1.2 μg; lane *5*, 1.6 μg; lane *6*, 2 μg; lane *7*, 2.4 μg; lane *8*, 2.4 μg + 1 mM EDTA. **b**, Effects of 1.2 μg of Dacin incubated at 37 °C with 25 μg of bovine fibrinogen for different times. Lane *1*, control; lane *2*, 5 min; lane *3*, 30 min; lane *4*, 1 h; lane *5*, 3 h; lane *6*, 6 h; lane *7*, 12 h; lane *8*, 24 h. **c**, Effects of the different inhibitors on Dacin’s fibrinogenolysis activity. Lane *1*, 5 mM EDTA; lane *2*, 5 mM EGTA; lane *3*, 5 mM 2-ME; lane *4*, 5 mM PMSF; lane *5*, negative control. **d**, Fibrin plate assay. *1*, crude venom (20 μg); *2*, Dacin (15 μg); *3*, Dacin (10 μg); *4*, saline. Every sample were inoculated into the wells in fibrin plate and incubated for 24 h at 37 °C

Dacin exhibited the strong fibrinogenolytic activities as it cleaved Aα and Bβ chains of bovine fibrinogen in the dose- and time-dependent manners. As shown in Fig. 3a, b, when 1.2 μg of Dacin was incubated with 25 μg of fibrinogen, Dacin preferentially hydrolyzed Aα chain followed by Bβ chain within 60 min, but did not hydrolyzed γ chain. The fibrinogenolytic activities of Dacin were completely inhibited by chelating agents (EDTA or EGTA), as well as *β*-mercaptoethanol. In addition, it was not inhibited by PMSF (Fig. 3c). Dacin also showed the mild fibrinolytic activities as indicated in fibrin plate assays (Fig. 3d).

### PLA_2_ and hemorrhagic activities

**Fig. 4:**
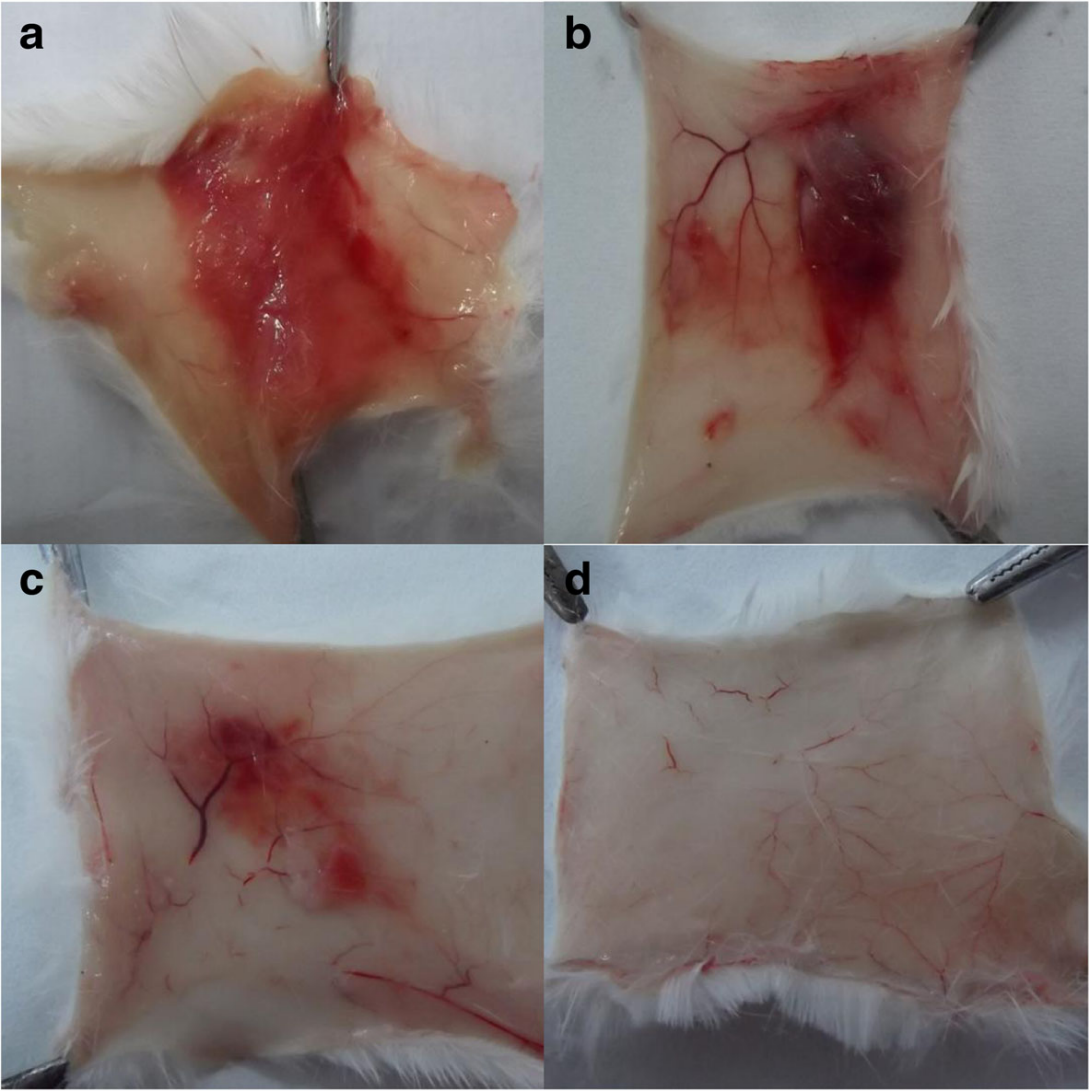
Hemorrhagic activities of Dacin on the mouse dorsal skin. Mice were injected intradermally in the dorsal skin with crude venom (**a**, 20 μg), or Dacin (**b**, 30 μg; **c**, 10 μg), or normal saline (**d**). After 2 h, the skin were removed and observed

In egg-yolk-suspension Petri dish assay, no cleared areas or transparent zones were developed from circumscribed Dacin depots (data not shown), and this indicated obtained Dacin sample had no PLA_2_ activity or to some extent meant that the obtained Dacin sample did not contain any amount of PLA_2_ component from *D. acutus* venom. Hemorrhagic activity was detected when Dacin was injected s.c. into mice (Fig. 4).

### Inhibitory effect on the contraction tension of mouse ileum smooth

**Fig. 5:**
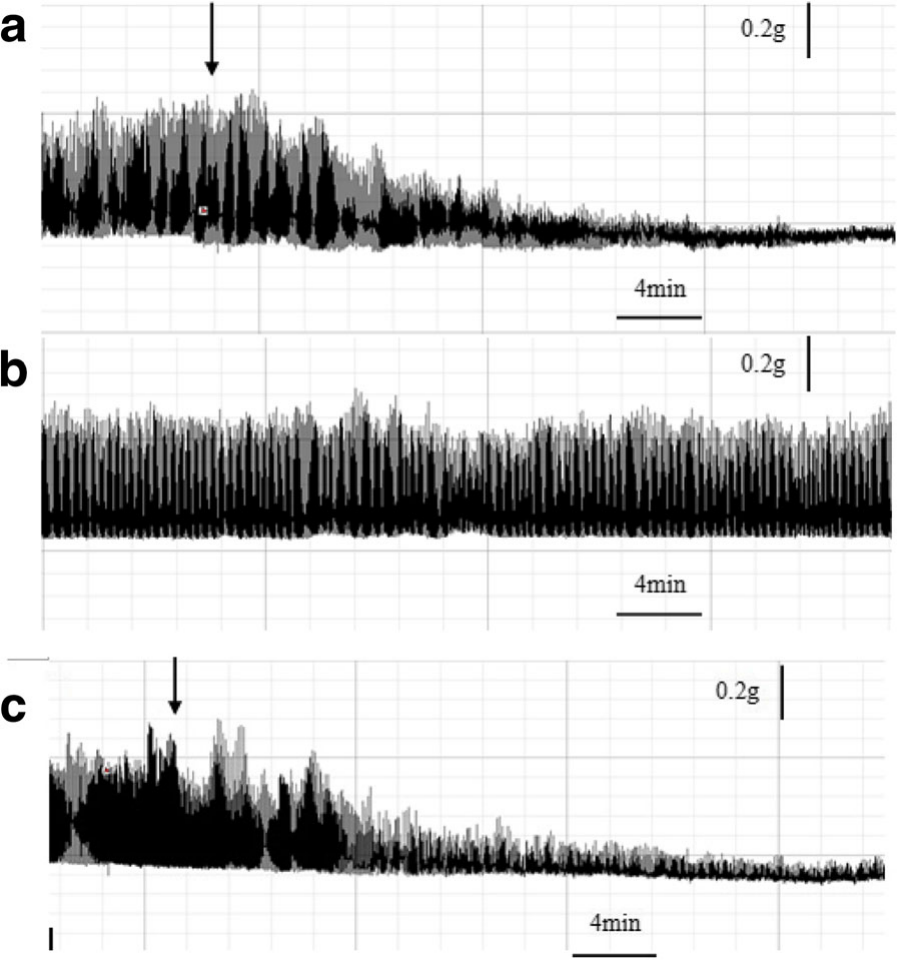
**a**, Trace showing the effect of Dacin (5 μg/ml) on the stimulated (70–100 V, 0.3 ms, 0.2 Hz) ileum smooth muscle preparation. *Arrow* indicates addition of Dacin. **b**, Control experiment without toxin or venom. **c**, Effect of crude venom (5 μg/ml) on the stimulated (70–100 V, 0.3 ms, 0.2 Hz) ileum smooth muscle preparation. *Arrow* indicates addition of venom

**Fig. 6:**
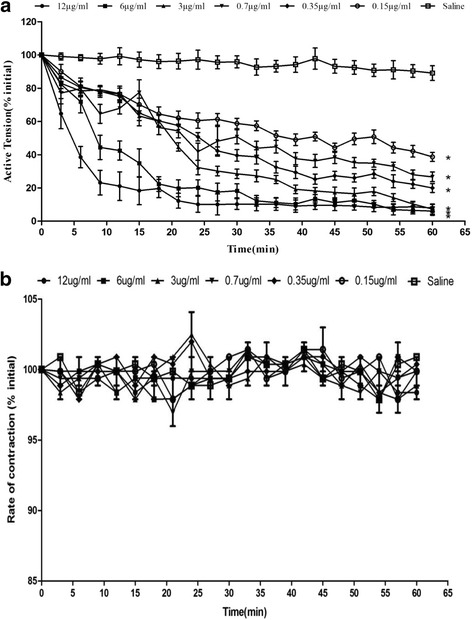
Responses of the various concentrations of Dacin (**a**) or boiled Dacin (**b**) (0.15, 0.35, 0.7, 3, 6, 12 μg/ml, *n*=3; saline control experiment, *n*=4) on the electrically stimulated (70–100 V, 0.3 ms, 02 Hz) ileum smooth muscle of mouse. The values are the Mean ± SEM counted as percent of initial. *P < 0.05 significantly different to saline control experiment

In Fig. 5, Dacin or the crude venom showed obvious inhibitory effects on the contraction tension of mouse ileum smooth muscle preparation. Dacin showed the significant time- and dose-dependent inhibitory effects in amplitude of active tension compared with normal saline (Krebs solution) (Fig. 6a). The most significant effect was observed at the higher concentrations of Dacin and the t_50_ blockade also exhibited the concentration-dependent manner (Table 1). In addition, Dacin’s inhibitory effect was irreversible because the spontaneous contraction could not be restored after washing (data not shown), and when Dacin was boiled at 100 °C for 5 min, its inhibitory effect was abolished, as Fig. 6b indicated that the boiled Dacin’s inhibitory effects on contraction tension of mouse ileum were insignificant when compared with control experiment.

**Table 1:** Time of causing 50% blockade (t_50_) and maximum inhibition rates (%) at 60 min time point in the different concentrations of Dacin on the contractive tension of mouse ileum

Dacin concentration (μg/ml)	Time of 50% blockade^a^ (min)	Maximum % in inhibition (at 60 min)
0.15	37 ± 3	60%
0.35	24 ± 3	64%
0.7	17 ± 9	77%
3	12 ± 3	83%
6	9 ± 2	91%
12	3 ± 1	94%

^a^Mean ± SEM, *n* = 3

## Discussion

A number of studies had been made on snake venoms and their isolated protein components for investigating the neurotoxic and myotoxic effects [24–32]. It was well-known that not only the neurotoxins and the myotoxins were extensively discovered as the main components in the venoms of Elapidae as well as Hydrophiidea, but also recently they were found as the minor components in the venoms of Viperidae and Crotalidae, especially Colubridae. For example, Harvey et al. used the chick biventer cervicis nerve-muscle and the phrenic nerve-diaphragm preparations of rat and mouse to assess the neurotoxic and the myotoxic effects of the venoms from eight species of snakes, respectively belonged to Elapidae, Viperidae and Crotalidae, and found that the venoms collected from the snakes of Elapidae, completely blocked neuromuscular transmissions and also caused myotoxic activities, however the viper venoms had slightly limited neurotoxic activities [11–13]. Afterwards, numerous studies indicated that the venoms of Elapid snakes contained a highly amount of neurotoxins, as well as some myotoxins, and the viper venoms had a large amount of haemotoxins and some slight neurotoxins [33–35].

In the venoms of most species of viper snakes, SVMPs are the most abundant components or haemotoxins [36]. SVMPs are able to interact with different targets that control hemostasis or relevant tissues related to essential physiological functions in prey and predators and give rise to the most evident effect, hemorrhage [37, 38]. The mechanisms of action of distinct SVMPs involve different targets as activation of coagulation Factor X [39], activation of Factor II [40], fibrino(gen)olytic activity [41], binding and damage of capillary vessels [42], among others. However, to our knowledge there are no papers to report SVMPs present the activity of inhibiting the contraction tension of mouse ileum.

Our preliminary test revealed that *D. acutus* venom showed the activity of inhibiting the contraction tension of mouse ileum. This clue led us in this study to focus on the active components, which could inhibit the contraction tension of ileum, in the venom of *D. acutus*. By three steps of chromatographies the active component, Dacin, was isolated and purified from venom of *D. acutus* (Fig. 1).

It was found that Dacin presented only one band on SDS-PAGE under reducing or non-reducing conditions (Fig. 2), and unique one peak in RP-HPLC. These results indicated that the obtained Dacin was homogeneous. Further, all the results of its biochemical and biological assays, MALDI-TOF-MS and Mascot analysis, fibrino(geno)lytic activity and metal-chelating agent’s inhibitory assays (Fig. 3), PLA_2_ activity assay, and hemorragic activity assays (Fig. 4), revealed that Dacin, without PLA_2_ activity, was a hemorragic SVMP, which belongs to P-I class in three classes (P-I, P-II, and P-III) of SVMP family [43, 44]. To our knowledge, a few of published papers reported the venoms of some vipers presented neurotoxic effects, for example, the venom of *Echis carinatus*, could inhibit the active tension of rabbit intestine smooth muscle [45], and the mild neurotoxicity was observed in severely envenomed Sri Lankan Russell’s viper (*Daboia russelii*) bites [12, 13]. In the current study, we observed that the venom of the five-pace viper (*D. acutus*) inhibited the contraction tension of mouse ileum (Fig. 5). It was evidenced that one SVMP component, Dacin, in the venom of *D. acutus* played a role of inhibiting the contraction tension of mouse ileum, besides it showed the time-dependent and concentration-dependent effects (Fig. 6, Table 1). Meanwhile, this inhibitory response of Dacin was irreversible. Although some PLA_2_s in snake venoms performed some pre-synaptic or post-synaptic effects [12, 13], in this study obtained Dacin sample from *D. acutus* venom had no PLA_2_ activity and Dacin’s activity of inhibiting the contraction tension of mouse ileum was not involved in the action of PLA_2_ constituent in *D. acutus* venom. These findings may provide a new insight for toxicological studies of SVMPs and the venoms of vipers, and give a reference for clinicians to treat the snake-bitten victims. However, whether there are other toxins, which may inhibit the contraction tension of mouse ileum, naturally in *D. acutus* venom or not, and what is the inhibitory molecular mechanism of Dacin, and so on, all these questions will be worthy to be studied in the future.

## Conclusion

In summary, for the first time the active component (Dacin, a SVMP) hat irreversibly inhibited the spontaneous contraction tension of mouse ileum has been isolated and identified in *D. acutus* venom. The findings not only may provide a new insight for toxicological researches on SVMPs and venoms of vipers, but also give a reference for clinicians to treat the snake-bitten victims. However, Dacin’s inhibitory molecular mechanism will be further studied in the future.

## Abbreviations

BSA: Bovine serum albumin
DEAE: Dicthylaminoethyl
EDTA: Ethylene diamine tetraacetic acid
EGTA: Ethylene glycol-bis(β-aminoethyl ether)-N,N,N′,N′-tetraacetic acid
HPLC: High performance liquid chromatography
MALDI-TOF-MS: Matrix-Assisted Laser Desorption/ Ionization Time of Flight Mass Spectrometry
PBS: Phosphate buffered saline
PLA2: Phospholipase A2
PMF: Peptide mass fingerprint
PMSF: Phenylmethanesulfonyl fluoride
SDS: Sodium dodecyl sulfate
SDS-PAGE: Sodium dodecyl sulfate-polyacrylamide gel electrophoresis
SVMPs: Snake venom metalloproteinases
SVSPs: Snake venom serine proteases
TFA: Trifluoroacetic acid

## References

[1] Qin GP (1998). China poisonous snake research.

[2] Zhao EM (2006). Snakes of China.

[3] Chen CC, Yang CM, Hu FR, Lee YC (2005). Penetrating ocular injury caused by venomous snakebite. Am J Ophthalmol.

[4] Li QB, Yu QS, Huang GW, Tokeshi Y, Nakamura M (2000). Hemostatic disturbances observed in patients with snakebite in south China. Toxicon.

[5] White J (2005). Snake venoms and coagulopathy. Toxicon.

[6] Qinghua L, Xiaowei Z, Wei Y, Chenji L, Yijun H (2006). A catalog for transcripts in the venom gland of the *D. acutus*: identification of the toxins potentially involved in coagulopathy. Biochem Biophys Res Commun.

[7] Markland FS, Swenson S (2013). Snake venom metalloproteinases. Toxicon.

[8] Hodgson WC, Wickramaratna JC (2006). Snake venoms and their toxins: an Australian perspective. Toxicon.

[9] Sajevic T, Leonardi A, Krizaj I (2011). Haemostatically active proteins in snake venoms. Toxicon.

[10] Fox JW, Serrano SM (2005). Structural considerations of the snake venom metalloproteinases, key members of the M12 reprolysin family of metalloproteinases. Toxicon.

[11] Harvey AL, Barfaraz A, Thomson E, Faiz A, Preston S (1994). Screening of snake venoms for neurotoxic and myotoxic effects using simple in vitro preparations from rodents and chicks. Toxicon.

[12] Silva A, Kuruppu S, Othman I, Goode RJ, Hodgson WC (2017). Neurotoxicity in Sri Lankan Russell's viper (*D. russelii*) envenoming is primarily due to U1-viperitoxin-Dr1a, a pre-synaptic neurotoxin. Neurotox Res.

[13] Kumar JR, Basavarajappa BS, Vishwanath BS, Gowda TV (2015). Biochemical and pharmacological characterization of three toxic phospholipase A2s from *D. russelii* snake venom. Comp Biochem Physiol C Toxicol Pharmacol.

[14] Tuladhar BR, Womack MD, Naylor RJ (2000). Pharmacological characterization of the 5-HT receptor-mediated contraction in the mouse isolated ileum. Br J Pharmacol.

[15] Okada T, Narai A, Matsunaga S, Fusetani N, Shimizu M (2000). Assessment of the marine toxins by monitoring the integrity of human intestinal Caco-2 cell monolayers. Toxicol in Vitro.

[16] Paton WD, Zar MA (1968). The origin of acetylcholine released from guinea-pig intestine and longitudinal muscle strips. J Physiol.

[17] Aniya Y, Sakanashi M, Noguchi K, Matsusaki K (1985). Heat stable protein with anticoagulant and smooth muscle contractile actions isolated from Habu (*Trimeresurus flavoviridis*) venom. Jpn J Pharmacol.

[18] Lowry OH, Rosebrough NJ, Farr AL, Randall RJ (1951). Protein measurement with the Folin phenol reagent. J Biol Chem.

[19] Laemmli UK (1970). Cleavage of structural proteins during the assembly of the head of bacteriophage T4. Nature.

[20] Rodrigues VM, Soares AM, Guerra-Sa R, Rodrigues V, Fontes MRM (2000). Strucutral and functional characterization of neuwiedase, a nonhemorrhagic fibrinogenolytic metalloprotease from *B. neuwiedi* snake venom. Arch Biochem Biophys.

[21] Habermann E, Hardt KL (1972). A sensitive and specific plate test for the quantitation of phospholipases. Anal Biochem.

[22] Kondo H, Kondo S, Ikezawa H, Murata R (1960). Studies on the quantitative method for determination of hemorrhagic activity of Habu snake venom. Jpn J Med Sci Biol.

[23] Nikai T, Kato C, Komori Y, Nodani H, Homma M, Sugihara H (1995). Primary structure of Ac1-proteinase from the venom of *D. acutus* (hundred-pace snake) from Taiwan. Biol Pharm Bull.

[24] Balhara KS, Stolbach A (2014). Marine envenomations. Emerg Med Clin North Am.

[25] Hart AJ, Smith AI, Reeve S, Hodgson WC (2005). Isolation and characterisation of acanmyotoxin-2 and acanmyotoxin-3, myotoxins from the venom of the death adder *Acanthophis sp. Seram*. Biochem Pharmacol.

[26] Kuruppu S, Isbister GK, Hodgson WC (2005). Phospholipase A2-dependent effects of the venom from the new Guinean small-eyed snake *Micropechis ikaheka*. Muscle Nerve.

[27] Lumsden NG, Banerjee Y, Kini RM, Kuruppu S, Hodgson WC (2007). Isolation and characterization of rufoxin, a novel protein exhibiting neurotoxicity from venom of the psammophiine, *Rhamphiophis oxyrhynchus* (Rufous beaked snake). Neuropharmacology.

[28] Lumsden NG, Fry BG, Ventura S, Kini RM, Hodgson WC (2005). Pharmacological characterisation of a neurotoxin from the venom of *Boiga dendrophila* (mangrove catsnake). Toxicon.

[29] Pawlak J, Mackessy SP, Sixberry NM, Stura EA, Le Du MH (2009). Irditoxin, a novel covalently linked heterodimeric three-finger toxin with high taxon-specific neurotoxicity. FASEB J.

[30] Petrova SD, Atanasov VN, Balashev K (2012). Vipoxin and its components: structure-function relationship. Adv Protein Chem Struct Biol.

[31] Tamiya N, Yagi T (2011). Studies on sea snake venom. Proc Jpn Acad Ser B.

[32] Venkatesh M, Prasad N, Sing T, Gowda V (2013). Purification, characterization, and chemical modification of neurotoxic peptide from *D. russelii* snake venom of India. J Biochem Mol Toxicol.

[33] Wickramaratna JC, Fry BG, Aguilar MI, Kini RM, Hodgson WC (2003). Isolation and pharmacological characterization of a phospholipase a 2 myotoxin from the venom of the irian jayan death adder (*A. rugosus*). Br J Pharmacol.

[34] Wickramaratna JC, Hodgson WC (2001). A pharmacological examination of venoms from three species of death adder (*A. antarcticus, A. praelongus and A. pyrrhus*). Toxicon.

[35] Wickramaratna JC, Fry BG, Hodgson WC (2003). Species-dependent variations in the in vitro myotoxicity of death adder (Acanthophis) venoms. Toxicol Sci.

[36] Casewell NR, Wagstaff SC, Wüster W, Cook DA, Bolton FM (2014). Medically important differences in snake venom composition are dictated by distinct postgenomic mechanisms. Proc Natl Acad Sci U S A.

[37] Moura-da-Silva AM, Butera D, Tanjoni I (2007). Importance of snake venom metalloproteinases in cell biology: effects on platelets, inflammatory and endothelial cells. Curr Pharm Des.

[38] Bernardoni JL, Sousa LF, Wermelinger LS, Lopes AS, Prezoto BC (2014). Functional variability of snake venom metalloproteinases: adaptive advantages in targeting different prey and implications for human envenomation. PLoS One.

[39] Siigur E, Tõnismägi K, Trummal K, Samel M, Vija H (2001). Factor X activator from *vipera lebetina* snake venom, molecular characterization and substrate specificity. Biochim Biophys Acta.

[40] Modesto JC, Junqueira-de-Azevedo IL, Neves-Ferreira AG, Fritzen M, Oliva ML (2005). Insularinase a, a prothrombin activator from *Bothrops insularis* venom, is a metalloprotease derived from a gene encoding protease and disintegrin domains. Biol Chem.

[41] Kamiguti AS, Slupsky JR, Zuzel M, Hay CR (1994). Properties of fibrinogen cleaved by jararhagin, a metalloproteinase from the venom of *Bothrops jararaca*. Thromb Haemost.

[42] Escalante T, Shannon J, Moura-da-Silva AM, Gutiérrez JM, Fox JW (2006). Novel insights into capillary vessel basement membrane damage by snake venom hemorrhagic metalloproteinases: a biochemical and immunohistochemical study. Arch Biochem Biophys.

[43] Fox JW, Serrano SMT (2008). Insights into and speculations about snake venom metalloproteinase (SVMP) synthesis, folding and disulfide bond formation and their contribution to venom complexity. FEBS J.

[44] Seo T, Sakon T, Nakazawa S, Nishioka A, Watanabe K, et al. Haemorrhagic snake venom metalloproteases and human ADAMs cleave LRP5/6, which disrupts cell-cell adhesions in vitro and induces haemorrhage in vivo. FEBS J. 2017;284:1657–71. 10.1111/febs.14066 28425175

[45] Savanur A, Ali SA, Munir I, Abbasi A, Alam M (2014). Pharmacological and biochemical studies on the venom of a clinically important viper snake (*Echis carinatus*) of Pakistan. Toxicon.

